# A Pilot Study of an In-Home Multicomponent Exergame Training for Older Adults: Feasibility, Usability and Pre-Post Evaluation

**DOI:** 10.3389/fnagi.2019.00304

**Published:** 2019-11-22

**Authors:** Manuela Adcock, Melanie Thalmann, Alexandra Schättin, Federico Gennaro, Eling D. de Bruin

**Affiliations:** ^1^Institute of Human Movement Science and Sport, Department of Health Sciences and Technology, ETH Zürich, Zurich, Switzerland; ^2^Division of Physiotherapy, Department of Neurobiology, Care Sciences and Society, Karolinska Institute, Stockholm, Sweden

**Keywords:** exergame, healthy aging, fall prevention, elderly, feasibility, usability

## Abstract

Aging is associated with sensory, motor and cognitive impairments that may lead to reduced daily life functioning including gait disturbances, falls, injuries and mobility restrictions. A strong need exists for implementing effective evidence-based interventions for healthy aging. Therefore, the aim of this study was to (i) evaluate the feasibility and usability of an in-home multicomponent exergame training and (ii) explore its effects on physical functions, cognition and cortical activity. Twenty-one healthy and independently living older adults were included (11 female, 74.4 ± 7.0 years, range: 65–92 years) and performed 24 trainings sessions (each 40 min) over eight weeks. The first part was conducted in a living lab (home-like laboratory environment), the second part at participants’ home. The multicomponent exergame included Tai Chi-inspired exercises, dance movements and step-based cognitive games to train strength, balance and cognition. Attendance and attrition rates were calculated and safety during training was evaluated to determine feasibility. Participants rated the usability of the exergame (System Usability Scale) and reported on their game experience (Game Experience Questionnaire). Physical and cognitive functions and cortical activity (resting state electroencephalopathy) were assessed pre and post intervention. Results showed a high training attendance rate for the living lab and the home-based setting (91.7 and 91.0%, respectively) with a rather high attrition rate (28.6%, six drop-outs). Half of the drop-out reasons were related to personal or health issues. System usability was rated acceptable with a mean score of 70.6/100. Affective game experience was rated favorable. Significant improvements were found for minimal toe clearance, short-term attentional span, and information processing speed (*p* < 0.05). No significant pre-post differences were found for cortical activity. To summarize, the exergame is generally feasible and usable for healthy older adults applied in an in-home setting and provides an overall positive emotional game experience. Nevertheless, flawless technical functionality should be a mandatory consideration. Additionally, the training might have potential positive influence on specific functions in older adults. However, the efficacy has to be evaluated in a future randomized controlled trial assessing the behavioral and neuroplastic changes in a larger population after a longer training period.

## Introduction

As life expectancy is increasing, the number of people aged 60 years and older is rapidly growing. According to the World Health Organization, the number of older adults aged 60 years and above was about 9% of the worldwide population in 2015 and will more than double to 22% by 2050 (National Institutes of Health, 2011). Considering the rapid growth in the elderly population, the maintenance and improvement of health and independence of older adults is important for social and economic reasons. A major focus should aim to prevent problems that cause health restrictions and morbidity, e.g., fall events (Masud and Morris, 2001; Rubenstein, 2006). Falling can lead to serious adverse consequences as injuries, movement restrictions, and part to full loss of independence (Peel, 2011). In addition to the physical consequences, falls can also lead to fear of falling resulting in further activity restriction, social isolation, feelings of helplessness, depression, and a general decrease in well-being (Jung, 2008; Peel, 2011). Considering the significant impact on individual lives and on healthcare costs, a strong need exists to develop and examine interventions that aim to support healthy aging and prevent falls in older adults.

In order to successfully support healthy aging and prevent falls, risk factors of impaired daily life functioning including falling have to be identified. Age- and lifestyle-associated degenerative changes in motor and sensory systems are linked to impaired daily life activities e.g., gait performance (Willett et al., 2006). The often described decline in muscle strength and loss of balance are discussed as potential risk factors for gait disturbances and falling (Rubenstein, 2006; Taylor et al., 2012). Exercise interventions aiming to improve physical functions, such as strength or balance, have been shown to reduce fall rates and risks in older adults (Sherrington et al., 2008; El-Khoury et al., 2013; Karlsson et al., 2013; Schoene et al., 2014). However, walking, as most daily life activities, requires physical and cognitive resources (Holtzer et al., 2006; de Bruin and Schmidt, 2010; Mirelman et al., 2012). A continuous interaction of physical and cognitive functions is mandatory for safe gait and intact daily life functioning in general (Hausdorff et al., 2005; Delbaere et al., 2010). Therefore, more recently increased attention is paid to cognition as a further aspect of fall prevention programs. The aging brain undergoes several structural and functional changes which can lead to a decline in cognitive functions (Rossini et al., 2007; Gunning-Dixon et al., 2009). The reduced cognitive resources might contribute to impaired daily life functioning and fall events due to an impaired physical-cognitive interplay. Thus, a combined physical-cognitive training is important for effective and holistic support of healthy aging and fall prevention (Pichierri et al., 2011; Bamidis et al., 2014; Eggenberger et al., 2015). Promising options for simultaneous training of physical and cognitive functions are video game-based physical exercises, or so-called exergames (de Bruin et al., 2010).

The term exergaming is a portmanteau composed of “exercise” and “gaming” and is defined as “any type of video game play requiring players’ whole body movements” (Gerling and Mandryk, 2014). More recently, exergames have gained increasing popularity in supporting healthy aging and fall prevention (Van Diest et al., 2013, 2016). This might be related to the technological development but also to the multifaceted potential of exergames. Poor adherence is described for numerous existing conventional exercise interventions (Choi et al., 2017). Such barriers, that hinder the attendance at physical activities and exercise interventions, can be related to personal (behavioral) factors such as motivation, personal beliefs, lack of time or feeling uncomfortable in social settings (e.g., in training classes) (Schutzer and Graves, 2004; Yardley et al., 2006; Franco et al., 2015). Furthermore, environmental factors including distance to an exercise facility, poor access to transportation options and participation costs may lead to low(er) exercise compliance as well (Schutzer and Graves, 2004; Yardley et al., 2006; Franco et al., 2015). Many of these barriers may be counteracted by exergames as they provide enjoyable and, therefore, motivating gameplay (Hoffmann et al., 2015; Pirovano et al., 2016). Furthermore, exergames can be applied in diverse settings. For example, the application in an in-home setting facilitates access without obstructions. Moreover, exergames can be adapted to specific purposes and target populations, which might be important when using interventions for public health and disease prevention. To summarize, advantages of exergames compared to conventional exercises are: (1) Exergames can imply simultaneous training of physical and cognitive functions (thereby being closer to daily life requirements) (Van Diest et al., 2013); (2) Users get motivated through an engaging and interactive training (Choi et al., 2017); (3) Training principles (e.g., feedback, progression, task variability) can be implemented in the exergame structure (Healy et al., 2014; Skjæret et al., 2016); (4) Exergame training can be conducted in diverse settings, e.g., at home. Recent studies have shown high acceptability of home-based exergame training for older adults after a mild injury and for elderly living in a nursing home (Valiani et al., 2017; Lauzé et al., 2018). Thus, developing an exergame for an in-home training solution might be a promising strategy to overcome some of the barriers for training attendance in older adults.

Considering the theoretical background from human movement science and neuropsychology together with the art of game design, we developed the Active@Home exergame to support healthy aging including fall prevention. Following a user-centered design approach, the needs and requirements of older adults as target users have been incorporated (Brox et al., 2017). The Active@Home exergame includes strength, balance, and cognitive training components and aims at motivating older adults toward a more active lifestyle. A first pilot-study explored the usability of the newly developed exergame prototype in a laboratory setting where training sessions were closely supervised. The results showed high usability of the exergame prototype and, furthermore, delivered some suggestions for improvements and adaptations in a next iterative development step. The information gathered in the first exploratory trial were, therefore, used to develop the exergame intervention for application at older adults’ homes by following a framework for the design and evaluation of complex interventions (Campbell et al., 2000). Therefore, the primary aim of this study was to evaluate the feasibility and usability of the modified Active@Home exergame in an in-home setting and to gain information from older adults about the training system. A secondary aim was to explore whether this intervention effects on physical and/or cognitive functions as well as on cortical activity at rest.

## Materials and Methods

### Study Design and Participants

This study is an exploratory trial, a phase II study according to Campbell and colleagues, using a single arm pre-post testing design (Campbell et al., 2000). Recruitment of participants was conducted in March 2018 through public advertisements in a local newspaper (Höngger Zeitung, Zurich, Switzerland) and through contacting the pensioner community ETH Zurich (PVETH, Zurich, Switzerland). Assessments and the first half of the intervention were performed at ETH Hönggerberg (Zurich, Switzerland). The second half of the intervention took place at participants’ home. The intervention period started in April 2018 and lasted until the beginning of June 2018. Measurements were conducted before and after the intervention. Ethical approval (protocol number EK 2018-N-07) was granted by the ETH Zurich Ethics Committee (Zurich, Switzerland). All participants were fully informed prior to participation and signed an informed consent form according to the Declaration of Helsinki before conducting any measurement.

For this trial, healthy and independent living older adults aged 65 years and above were recruited. To evaluate the eligibility of the potential participants, they were screened using the Mini Mental State Examination (MMSE) to assess cognitive status. In addition, the participants completed a health questionnaire including anthropometric data and questions about their health, medical history and physical activity level. Participants fulfilling all of the following inclusion criteria were eligible for the study: (1) age ≥ 65 years, (2) living independently, (3) healthy (self-reported), (4) able to stand at least for 10 min without assistance, (5) access to a TV with HDMI connection. Participants exhibiting at least one of the following criteria were excluded from the study: (1) mobility impairments that prevent from training participation, (2) severe and uncontrolled health problems (e.g., recent cardiac infarction, uncontrolled diabetes or hypertension), (3) orthopedic disease that prevents from training participation, (4) neurological disease (e.g., history of stroke or epilepsy, Parkinson’s disease), (5) Alzheimer disease or other forms of dementia, (6) acute severe, rapidly progressive or terminal illness, (7) cognitive impairments (MMSE ≤ 23 points), (8) intake of any psychoactive substances (e.g., neuroleptics, antidepressants), (9) high alcohol, caffeine or nicotine consumption. The minimal intended study sample size of 20 participants starting with the training program was based on similar feasibility studies (Wuest et al., 2014; Nawaz et al., 2016), the “rule of 12” for continuous variables (Moore et al., 2011) and the expected compliance with the intervention (Nyman and Victor, 2012). The “rule of 12” recommends to include at least 12 participants for pilot studies for providing valuable preliminary information (Moore et al., 2011).

### Exergame Intervention

**FIGURE 1:**
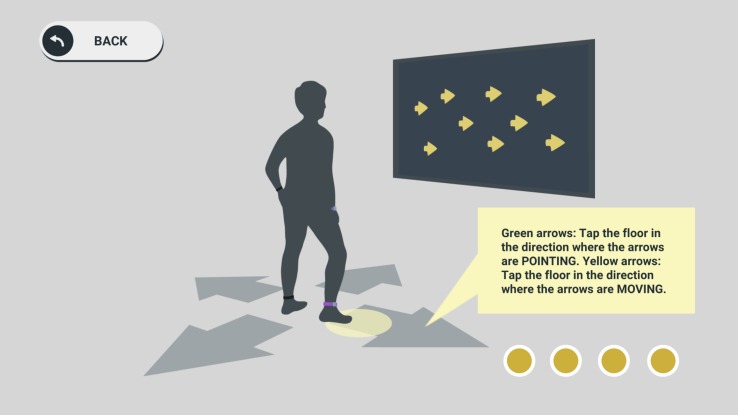
Step-based cognitive training. The Active@Home exergame included step-based exercises to train specific cognitive functions (a task for inhibition training is shown in the picture). By stepping forward, backward, and to the right or left side, these games were played and controlled. The IMUs worn at ankles evaluated the stepping performance.

The hardware of the Active@Home exergame consists of four inertial measurement units (IMUs) providing both accelerometer and gyroscope assessments. For movement evaluation, participants wore the IMUs at wrists and ankles attached with a silicone slap band. The IMUs were connected via Bluetooth to a HDMI dongle that was inserted into a television (TV) and ran the exergame software. The game interface was presented on the TV screen. The story of the exergame was about traveling in Europe and to train in several different cities (London, Paris, Amsterdam, Rome, Porto, and Zurich). Exercises were instructed by a cartoon-based instructor and were accentuated with background music (Tabei et al., 2017). The training content of the Active@Home exergame was based on current recommendations for exercises to prevent falls in older adults (American Geriatrics Society, British Geriatrics Society, and American Academy of Orthopaedic Surgeons Panel on Falls Prevention, 2001; Nelson et al., 2007; Chodzko-Zajko et al., 2009; Panel on Prevention of Falls in Older Persons et al., 2011; Sherrington et al., 2011; Bamidis et al., 2014). The exergame included Tai Chi-inspired exercises, dance movements as well as step-based cognitive games aiming to train strength, balance, and cognition, respectively. Tai Chi-based training has been discussed as a suitable exercise for fall prevention (Hu et al., 2016; Zhao and Wang, 2016). Tai Chi is a functional training involving bilateral and multidirectional movements (Xu et al., 2008), requiring whole body coordination (Hackney and Wolf, 2014), and improving core and lower limbs muscle strength; e.g., m. iliopsoas, m. quadriceps femoris and m. tibialis anterior (Solloway et al., 2016). In the Active@Home exergame, four to five Tai Chi levels were implemented in each city with increasing difficulty. In each Tai Chi level, the participant performed three series of about 10 exercise repetitions, with a rest of 20 s between series. This resulted in a duration of 2–3 min for each Tai Chi level. During the 20 s rest between series, participants were asked questions about the city in which they were exercising to entertain them. The initial positions of the Tai Chi-inspired exercises included squats, plies, lunges and single-leg stances. Furthermore, the Active@Home exergame contained dance exercises based on several different dancing styles (Bachata, Salsa, Cha-Cha-Cha, Waltz, Jive, and Disco Fox). Conducting rapid and well-directed steps has been shown to be an effective training for fall prevention (Kattenstroth et al., 2013; Okubo et al., 2016). In each city of the exergame, one dancing style was trained in three levels of difficulty. Each dance level lasted around 3 min. Additionally, step-based cognitive games were included in the Active@Home exergame focusing on specific cognitive functions such as selective attention, divided attention, mental flexibility, inhibition/interference control, and working memory. Deficits in these cognitive functions contribute to gait disturbances and falls (Amboni et al., 2013). By stepping forward, backward, and to the right or left side, the cognitive games were played and controlled (Figure 1). Each city of the exergame included three different step-based cognitive games, each game lasting between 1 and 2 min. Moreover, the exergame implemented some basic training principles (feedback, optimal load, progression, variability) which are important for effective training and for reaching the training goal of improvements in physical and cognitive functioning (Healy et al., 2014). A feedback system was included with a real-time color code for achieved performance (red color = “bad performance”; orange color = “moderate performance”; green color = “good performance”) and performance scores during and after each exercise. In the cognitive games, the visual feedback system described above was augmented with an auditory feedback system providing sounds to indicate the correctness of the answer. To ensure optimal load and progression, several difficulty levels for Tai Chi-inspired and dance exercises were developed in each city. Progression was reached through more complex movements in the Tai Chi-inspired exercises (e.g., additional arm movements, upper body rotations, increased range of motion, longer time in unstable position) and through additional weights (e.g., filled water bottles), while faster and more complex motion sequences were performed in dance exercises. To increase difficulty in step-based cognitive training, more complex games were used requiring e.g., faster reaction times or memorization of additional information. Moreover, the training exercises got more challenging from city to city with a predefined city order of progressive difficulty. Training variability was guaranteed through a high diversity of exercises in the different cities of the exergame.

From April to June 2018, each participant performed 24 training sessions in 8–10 weeks (a maximum of two weeks holiday interruption was allowed). Each week, three training sessions were performed, which were scheduled individually from Monday to Friday with a guideline of no more than one training session per day. Each session consisted of 40 min training with the Active@Home exergame including 15 min of Tai Chi-inspired training, 15 min of dance exercises, and 10 min of step-based cognitive games. It was recommended to play through all levels of one city before switching to the next city whereas the order of the cities was predefined based on progressive difficulty. Additionally, before changing the level, one level should have been trained at least in two sessions. The current difficulty level should always provide an optimal challenge avoiding under- or overload. The training intensity was thereby individually adapted to target a moderate training intensity level (Chodzko-Zajko, 2014). Intervention characteristics as frequency, duration, and training intensity were based on recommendations for fall prevention in elderly (Nelson et al., 2007; Paterson et al., 2007; Chodzko-Zajko et al., 2009) and on studies showing positive training effects of exergame training in older adults (Law et al., 2014).

Participants trained 3–4 weeks in the home-like living lab at ETH Hönggerberg (Zurich, Switzerland) and 4–5 weeks at their home. The training sessions in the living lab were supervised by two postgraduate students supporting the participants if needed and systematically observing them while using and training with the exergame. During the intervention period at home, participants were called weekly to provide help when needed.

### Primary Outcomes

A mixed method approach in form of a combination of quantitative and qualitative data collection was used to evaluate the primary outcome of feasibility and usability of the Active@Home exergame (Nawaz et al., 2016). Questionnaires were completed by participants after their last home training session of the intervention period (post-measurement).

#### Feasibility

Feasibility was assessed through compliance with the training intervention as well as by evaluation of the safety during training. An attendance protocol was used to record the number of performed training sessions. During the intervention period in the living lab, the protocol was filled in by the supervisors after each training session, whereas participants were instructed to protocol the training sessions during the intervention period at home. Additionally, during weekly calls by one of the supervisors, the protocoling procedure was checked. The adherence rate was calculated using the number of performed training sessions as percentage of the maximum possible training sessions (De Bruin et al., 2011; Wuest et al., 2014). A 70% attendance rate (17 visited out of 24 total training sessions planned) was considered “being adherent” to the training program (De Bruin et al., 2011; Nyman and Victor, 2012). The adherence rate was calculated for the two intervention periods (living lab, home) separately and in total. For attrition, the number of participants lost during the trial was recorded (drop-outs) and calculated as a percentage of the total sample size. Considering the median rate for attrition in fall prevention interventions for clinical trials, a 10% attrition rate (two drop-outs) was regarded acceptable (Nyman and Victor, 2012). Drop-outs were not considered in the calculation of the adherence rate. Moreover, reasons for non-adherence and drop-outs were recorded.

After the intervention, participants answered five questions related to safety including dizziness or pain during training, “critical moments” e.g., tripping, slipping or swaying, fear of falling or fall events. Safety questions were answered with “yes” or no” (in case of dizziness/pain also rating of level).

#### Usability

The System Usability Scale (SUS) is a validated and reliable scale for evaluating subjective usability of newly developed devices and systems and includes 10 items rated on a 5-point Likert scale (0 = “strongly disagree” to 4 = “strongly agree”) (Brooke, 1996; Vaziri et al., 2016). The sum of all item scores was multiplied with 2.5 and led to the SUS score theoretically ranging between 0 and 100. Higher scores indicate better usability (Brooke, 1996). Based on the verbal categorization rate of Bangor (Bangor et al., 2009), a SUS score ≥ 70 was expected for an “acceptable system”. An additional question was added at the end of the SUS, asking participants about their general opinion of the Active@Home exergame. This question was also rated on a 5-point Likert scale (0 = “I don’t like it” to 4 = “I like it a lot”) and the mean was calculated over all participants.

The Game Experience Questionnaire (GEQ) assesses seven categories of subjective game experience (competence, immersion, flow, tension, challenge, negative affect, and positive affect) (Hoffmann et al., 2015; Chesham et al., 2017). *Competence* implies feelings of being successful, strong or skillful in the game. *Immersion* includes the interest and pleasure of a player in the game. *Flow* summarizes the feelings of being deeply concentrated and absorbed, forgetting time, and losing connection to the world outside the game. *Tension* includes feelings of annoyance, frustration, and pressure. *Challenge* implies feelings of being stimulated and challenged. *Negative affect* summarizes feelings related to a bad mood and boredom, whereas *positive affect* includes feelings of happiness and enjoyment. The GEQ has been applied in several studies evaluating game experience of exergames for elderly (Liukkonen et al., 2015; Merriman et al., 2018). The GEQ core module, which was used in this study, includes in total 42 items rated on a 5-point Likert scale (0 = “not at all” to 4 = “extremely”). It was analyzed by calculating the average rating for each of the aforementioned categories (Ijsselsteijn et al., 2008). Two categories involved only negative coded items (tension and negative affect) leading to reverse evaluation (Chesham et al., 2017).

Observations by supervisors and feedback by participants were recorded on a usability protocol. The usability protocol was structured in six categories: (1) functionality and interaction with the system, (2) IMUs, (3) design, (4) training principles, (5) exercises, and (6) emotions, each category separated in positive and negative aspects. The participants were instructed (but not forced) to “think aloud” and mention all thoughts that came to their mind during exergame training (Lewis and Rieman, 1993). Their comments and the observations by the supervisors (during the intervention period in the living lab) were recorded in writing on the usability protocol. Furthermore, the protocol included feedback from participants collected during weekly calls (during the intervention period at home).

### Secondary Outcomes

As secondary outcomes, physical and cognitive functions as well as electroencephalographic cortical activity were measured before and after the intervention period (pre-measurement and post-measurement).

#### Physical Functions

Parameters of gait kinematics (speed, cadence, stride length, and minimal toe clearance) were assessed using the Physilog^®^5 IMU (Gait Up Sàrl, Lausanne, Switzerland), which has been shown to reliably and validly measure gait performance (Aminian et al., 1999; Dubost et al., 2006; de Bruin et al., 2007). The Physilog^®^5 IMUs were attached to the top of the right and left forefoot of participants using elastic straps. For further analysis, data was transferred to the computer via USB port. A walking protocol involving at least 50 gait cycles was applied (König et al., 2014). Participants walked a straight distance of 80 m under two conditions: (1) single-task condition (ST): participants were instructed to walk at preferred speed without talking; (2) dual-task condition (DT): participants had to walk at preferred speed and simultaneously count backwards (cognitive task) in steps of seven from a randomly given number between 200 and 250. In this condition, participants were asked to count out loud and perform both tasks concurrently and not to prioritize one task above the other. This is a common method to measure multitasking capabilities (Eggenberger et al., 2015; Schättin et al., 2016). Two walking steps for initiation and termination were discarded in order to analyze steady state walking (Jian et al., 1993). Speed [m/s], cadence [steps/min], stride length [m], and minimal toe clearance [cm] were evaluated and expressed as mean values of both legs in the two walking conditions. For each parameter, the dual-task cost (DTC) of walking was calculated as a percentage of loss of the DT relative to the ST condition according to the formula: DTC [%] = (ST – DT)/ST × 100 (McDowd, 1986).

To assess static balance, a subtest of the Short Physical Performance Battery (SPPB) was applied (Guralnik et al., 1994; Guralnik et al., 2000). The SPPB allows an objective and valid assessment of lower extremity functioning in elderly (Guralnik et al., 1994; Guralnik et al., 2000). The balance test of the SPPB includes the following tasks: standing in (1) feet side-by-side position, (2) semi-tandem stance, and (3) full-tandem stance. Each position should be held unsupported for 10 s. With a total score ranging from 0 (“not able to complete the tasks”) to 4 (“good balance function”), the performance can be evaluated. In line with previous studies (Eggenberger et al., 2015, 2016), we extended the balance test with two additional tasks to avoid ceiling effects. The first additional task was a 20 s single-leg stance (with preferred leg) where two points were achieved for reaching 20 s, one point for 10–20 s and zero points for <10 s. The second additional task was a single-leg stance (with preferred leg) with eyes closed where one point was assigned for every 5 s of successful task achievement. Three trials were conducted for each additional task whereas the best trial counted. For the extended version of the subtest, the maximum point score is unlimited with higher scores meaning better balance functioning. The total score of the extended subtest was calculated for the analysis.

Dynamic balance was assessed with the Y-Balance Test^TM^ (YBT^1^, Danville, VA, United States), a valid and reliable evaluation tool (Plisky et al., 2009) often used in rather active populations (Plisky et al., 2009; Shaffer et al., 2013) and recently applied in several studies with older adults (Lee et al., 2015; Shin and An, 2015; Donath et al., 2016). The testing kit consists of a stance platform to which three PVC bars are attached in the anterior, posteromedial, and posterolateral reaching directions (the posterior bars are positioned at 135 degrees from the anterior bar with 45 degrees between both posterior bars). The participants were asked to push a target along the bars in every direction with one foot (barefoot), while standing on the other foot on the stance platform with hands on the pelvis. Thereby, the maximal reaching distance in every direction can be measured. When the personal limit was reached, participants had to return to the starting position. For the purpose of familiarization, four practice trials were performed for each foot and direction. After a short break, three testing trials were accomplished using the following order: left anterior, right anterior, left posteromedial, right posteromedial, left posterolateral, and right posterolateral. Mean values of reaching distances were calculated over all testing trials for every direction and each foot. A testing trial was classified as invalid when participants (1) failed to maintain unilateral stance on the platform, (2) failed to maintain foot contact with the target while moving the target, (3) used the target for stance support, or (4) failed to return to the starting position. Invalid testing trials were required to be repeated. For data analysis, the final score was calculated by taking the sum of the mean maximum reaching distances in each direction and for each foot (six values in total) divided by three times the sum of right and left lower limb length, and then multiplied by 100. With this calculation, the averaged maximal reaching distance was normalized to the limb length of both legs (antero-superior iliac spine to the most distal part of the medial malleolus) and expressed as percentage of the lower limb length. Due to safety reasons, only participants who reached 20 s of single-leg stance in the extended version of the SPPB balance test were eligible for the YBT.

The Senior Fitness Test (SFT), a.k.a the Fullerton Fitness Test, was used to evaluate functional fitness of the participants (Rikli and Jones, 1999). In order to assess functional lower body strength and aerobic endurance, two subtests of the SFT test battery were chosen: the 30 s chair rises test and the 2 min stepping test. In the 30 s chair rises test, the participants had to cross their arms in front of the chest and perform as many full “chair rises” as possible in 30 s. A full chair rise was defined as sitting down on a chair and standing up, ending in an upright position again. The number of completed full chair rises in 30 s was counted. In the 2 min stepping test, the participants were asked to alternatingly step with both legs as many times as possible in 2 min while reaching a predefined individual height with the knees. This threshold height was calculated by means of the height from the floor to the middle of the thigh (midway between the iliac crest and the upper patella). For the analysis, the number of steps with the starting leg was counted during the 2 min whereas a step was valid only when the knee was reaching the required height.

#### Cognitive Functions

To assess cognitive functions, one computer-based test and three paper-pencil tests were used. The Test of Attentional Performance (D-TAP 2.3 VL, PSYTEST, Psychologische Testsysteme, Herzogenrath, Germany) is a computerized test battery to validly assess various attentional and executive functions (Zimmermann and Fimm, 2002). For this study, the subtest *Divided attention* was chosen to evaluate the capacity of dividing the attentional resources to stimuli in multiple modalities. The test was performed on a computer using an additional answer button and was preceded by a short familiarization session. Visual and acoustic signals were presented to the participants who had to react only to specific stimuli. Details about the protocol can be found elsewhere (Zimmermann and Fimm, 2002). Reaction times [ms], number of errors, and omissions were assessed.

The Trail Making Test (TMT) is a widely used, valid and reliable neuropsychological paper-pencil test to assess mental flexibility (Reitan, 1958; Corrigan and Hinkeldey, 1987; Bowie and Harvey, 2006). In the first part of the test (TMT A), participants had to connect randomly allocated, encircled numbers from 1 to 25 in ascending order as fast as possible. In the second part of the test (TMT B), the stimuli comprise encircled letters and numbers. The randomly allocated numbers and letters have to be connected in ascending numerical and alphabetical order alternatingly as fast as possible (e.g., 1 – A – 2 – B – 3 – C – …). In both parts, a short practice session was conducted. Time [s] for completing the tasks was recorded and errors were counted.

To assess response inhibition and interference control, the Victoria Stroop Test (VST) was used, a tool to validly and reliably measure executive functions (Strauss et al., 1991; Strauss et al., 2006; Troyer et al., 2006). The VST compromises three parts: (1) VST 1: naming the color of dots (red, blue, green, or yellow), (2) VST 2: naming the color of neutral words (e.g., words like “when” or “hardly” colored in red, blue, green, or yellow), and (3) VST 3: naming the color of “color words” printed in incongruent colors (e.g., word = red while word color = blue, etc.). In VST 3, interfering information is provided which requires interference control and response inhibition; the fast and automatic response of reading the words has to be inhibited and a more effortful color-naming response has to be produced (Bayard et al., 2011). Each part contains 24 stimuli. Performance time [s] was recorded for each task and errors were counted.

Further cognitive performance was evaluated with two subtests of the Wechsler Memory Scale-Revised (WMS-R) (Wechsler, 1987; Kent, 2013). The first subtest of the WMS-R, the digit forward task, assessed the short-term attention span and information processing speed (Wechsler, 1987; Hester et al., 2004). Participants had to remember and repeat digit sequences, which were read out loud by the tester, in the correct order. The first two sequences consisted of three digits, and afterwards, the sequence was extended with an additional digit for another two trials, and so on, until a maximum sequence length of eight digits was reached. The second subtest of the WMS-R, the digit backward task, was used to evaluate working memory capacity (Wechsler, 1987). Participants had to repeat the digit sequences in reversed order. Initially, the sequence consisted of two digits with the same extending procedure as described above, whereas the maximum sequence length was seven digits for this task. For every correct replication of a digit sequence, one point was scored, summing up to a total point score for each subtest (Peña-Casanova et al., 2009).

#### Cortical Activity and Analysis

Resting state EEG activity was acquired while the participants were seated comfortably for 5 min with eyes closed. EEG was recorded at 500 Hz sampling rate, using a 20-channels dry-electrodes cap (ENOBIO 20, Neuroelectrics, Barcelona, Spain) placed according to the international 10–20 system (Jasper, 1958) and referenced using the Driven-Right-Leg (DRL)/Common Mode Sense (CMS) technique (two external electrodes placed on either side of the left earlobe with an ear-clip). Before electrode placement on the forehead and earlobe, the skin was prepared with an abrasive and conductive gel (H + H Medizinprodukte GbR, Münster, Germany).

EEG data analysis was performed using custom scripts written in MATLAB R2017b (The Mathworks, Natick, MA, United States) and using the EEGLAB 14.1.0b open source toolbox (Delorme and Makeig, 2004). EEG data was first high-pass filtered [zero-phase Hamming windowed sinc FIR, cut-off frequency (−6 dB) 0.5 Hz, passband edge 1 Hz, transition bandwidth 1 Hz, order 1651] and subsequently low-pass filtered [zero-phase Hamming windowed sinc FIR, cut-off frequency (−6 dB) 45 Hz, passband edge 40 Hz, transition bandwidth 10 Hz, order 167]. Further analysis was performed solely to seven parieto-occipital EEG electrodes (Pz, P3/4, P7/8, and O1/2), since this cortical area is widely used to reliably detect individual alpha frequency (IAF) (Grandy et al., 2013; Corcoran et al., 2018). Channel rejection was performed using the automatic procedure supplied by the clean_rawdata EEGLAB extension by taking into account whether the correlation of a channel to a reconstruction of it based on other channels, in a given time window, was less than 0.4, and whether a channel was flat for more than 5 s. On average, ∼98% of the parieto-occipital channels in the pre-measurement EEG recordings remained for further analysis (σ: ∼5%; range: ∼86–100%) and ∼95% (σ: ∼9%; range: ∼71–100%) in the post-measurement EEG recordings. Artifactual data points were rejected when their amplitude was higher than ±75 μV within a 500 ms width time window as detected by the trimOutlier EEGLAB plugin. On average, ∼5% of data was rejected in the pre-measurement EEG recordings (σ: ∼11%; range: ∼0–43%) and ∼6% (σ: ∼14%; range: ∼0–17%) in the post-measurement EEG recordings. Afterwards, two IAF measures were estimated: peak alpha frequency (PAF) and center of gravity (CoG), by means of the resting IAF v1.0 open source package available from https://github.com/corcorana/restingIAF. This allowed a fully automatic and reliable strategy to determine IAF estimates during resting state EEG recordings, of which a more detailed and extensive description can be found elsewhere (Corcoran et al., 2018; Cross et al., 2018). Briefly, one-sided channel-wise power spectral density (PSD) was first calculated in the 1–40 Hz frequency range by the Welch’s modified periodogram method, using a 2048 sample (∼4 s) Hamming window (50% overlap) across segments (frequency resolution = 0.244 Hz) and normalized by dividing each PSD channel estimate (within the passband) by the mean spectral power. Then, each PSD estimate was smoothed using a Savitzky–Golay filter with frame length equals to 11 frequency bins and polynomial degree of five. From the smoothed PSD and within an *a priori* defined alpha frequency band (7–13 Hz), evident frequency peaks were detected and IAF estimates from spectral peaks’ boundaries were computed. Using the first derivative to detect spectral peaks seemingly yields true estimates compared to simply searching for maximal values within a predefined alpha frequency band (Grandy et al., 2013). Finally, IAF estimates were computed by averaging the obtained spectral peaks estimates across channels. The minimum number of valid channels necessary to estimate IAF was set to one, given the relatively low-density parieto-occipital EEG channels used for this analysis. Additionally, spectral power within the alpha frequency band was calculated by averaging in each participant the PSD estimates of all the included channels, and then summing the obtained channels mean power across the alpha frequency band. Alpha spectral bandwidth was defined as the individual PAF ± 2 Hz.

### Other Outcome Measures

The participants rated their current training motivation on a Visual Analog Scale (1 = unmotivated lethargic smiley to 5 = motivated happy smiley) before each training session. The average training motivation was separately calculated for the training period in the living lab and at home. Subjective exercise intensity was reported after each training session during the whole intervention period. The participants estimated their perceived exertion on the 20-point Borg scale that ranges from 6 to 20 (6 = “less than very light”, 20 = “more than very hard”) for Tai Chi-inspired and dance exercises, respectively (Borg, 1982). To target a moderate training intensity, Borg scale ratings in the range of 12 to 14 were expected (Borg, 1982). Furthermore, objective exercise intensity was assessed by heart rate (HR) measurements during at least three training sessions performed in the living lab. A Polar M400 (Polar Electro Oy, Kempele, Finland) was used to record HR for Tai Chi-inspired and dancing training separately. The individual maximal HR (HR_*max*_) was estimated with the formula: HR_*max*_ = 220 – age. The average HR while training (HR_*train*_) was compared to the individual estimated HR_*max*_ by calculating the HR_*train*_ as percentage of the HR_*max*_. To target a moderate training intensity, an average HR_*train*_ about 60% of HR_*max*_ was expected (Nelson et al., 2007; Chodzko-Zajko et al., 2009).

### Statistical Analysis

SPSS 23.0 for Windows (SPSS Inc, Chicago, IL, United States) was used for statistical analysis. Descriptive statistics were generated for all variables. Following a conservative approach and due to non-normality of some of the data, confirmed by both Shapiro–Wilk test and Q-Q-plots, a non-parametric testing strategy was used. Intragroup differences between pre- and post-measurements were analyzed by Wilcoxon signed-rank test. A significance level of α = 0.05 was applied. Correlational effect sizes (r), according to the following equation: r = z/√(n1 + n2) with n = sample size, n1 = n at pre-measurement and n2 = n at post-measurement, were calculated in MS Office Excel (version 2016) and reported according to Cohen (Cohen, 1988): an effect size of *r* = 0.10 indicates a small effect, *r* = 0.30 a medium effect, and *r* ≥ 0.50 a large effect. For pre- and post-measurement comparisons, drop-outs were excluded from analysis (per-protocol analysis). The analysis does not consider intention-to-treat analysis because of a clear description of the drop-out reasons (Moher et al., 2010). Moreover, only participants who reached 70% of the maximal possible training sessions were included in the pre-post-comparison.

## Results

**FIGURE 2:**
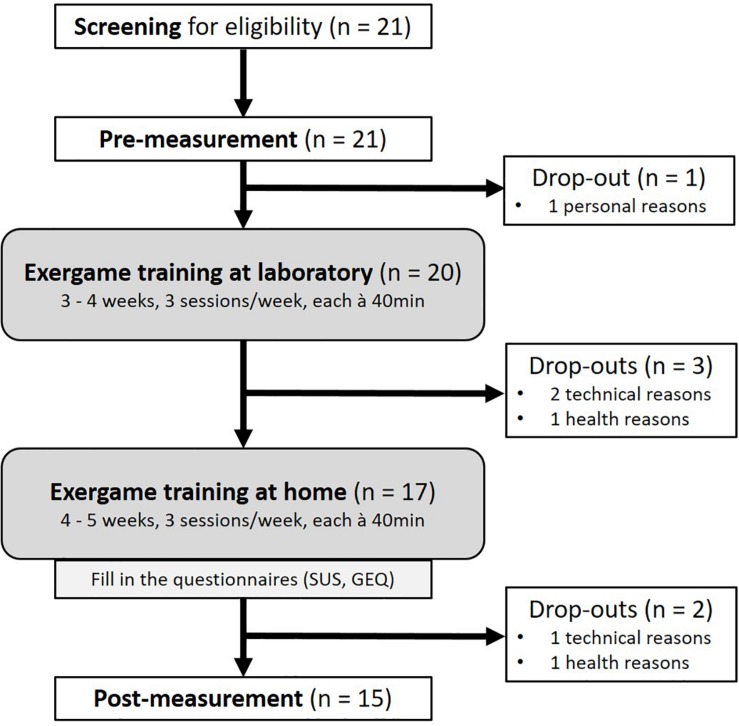
Study flow chart. Screening of participants for eligibility included an assessment of cognitive and health state. Physical and cognitive functions as well as brain activity were measured at pre- and post-measurement. Assessments and the first half of the intervention were performed at the living lab of ETH Hönggerberg (Zurich, Switzerland), the second half at participants’ home. Questionnaires assessing usability and game experience were filled in after the training period. Technical drop-out reasons included software problems, unstable IMU connections and inappropriate movement evaluation. Other drop-out reasons were related to injuries, sudden illness and family affairs. SUS, System Usability Scale; GEQ, Game Experience Questionnaire.

Twenty-one participants signed informed consent and were included in the study. Fifteen participants completed the 8-week training intervention. Six participants prematurely terminated study participation. The study flow chart is presented in Figure 2. Table 1 summarizes the demographic characteristics and screening measures of the drop-outs and the remaining participants separately. The drop-outs were comparable to the rest of the participants regarding their characteristics except of age (*U* = 75.50, *p* = 0.017) and self-evaluated balance at baseline [χ^2^(df) = 9.64(3), *p* = 0.022].

**TABLE 1:** Demographic characteristics of participants and screening values.

**Participant characteristics**	***n* = 21**	***n* = 6 (drop-outs)**
Age in years	74.4 ± 7.0 (65–92)	80.2 ± 7.1 (70–92)
Weight [kg]	73.2 ± 19.8 (42–120)	66.0 ± 20.0 (42–95)
Height [cm]	168.0 ± 9.4 (155–187)	168.0 ± 12.4 (155–187)
Education in years	14.1 ± 4.2 (4–20)	13.8 ± 6.1 (4–20)
MMSE Score	29.0 ± 1.6 (24–30)	27.8 ± 2.6 (24–30)
Female [n,%]	11 (52.4)	2 (33.3)
**Fear of falling [*n*,%]**		
Never	17 (81.0)	6 (100.0)
Sometimes	3 (14.3)	0 (0.0)
Often	1 (4.8)	0 (0.0)
Always	0 (0.0)	0 (0.0)
**Number of falls during last month^+^ [*n*,%]**		
Never	18 (85.7)	6 (100.0)
Once	2 (9.5)	0 (0.0)
More than once	1 (4.8)	0 (0.0)
**Self-evaluation of health state [*n*,%]**		
Very good	5 (23.8)	1 (16.7)
Good	12 (57.1)	2 (33.3)
Medium	4 (19.0)	3 (50.0)
Bad	0 (0.0)	0 (0.0)
**Self-evaluation of balance [*n*,%]**		
Very good	3 (14.3)	3 (50.0)
Good	9 (42.9)	1 (16.7)
Medium	7 (33.3)	2 (33.3)
Bad	2 (9.5)	0 (0.0)
**Self-evaluation of muscle strength [n,%]**		
Very good	2 (9.5)	1 (16.7)
Good	12 (57.1)	4 (66.7)
Medium	7 (33.3)	1 (16.7)
Bad	0 (0.0)	0 (0.0)

*Data are mean values ± standard deviations (ranges) or number of participants per category (absolute and relative frequencies). MMSE, Mini Mental State Examination. ^+^Self-stated.*

### Primary Outcome: Feasibility and Usability

The adherence rate over the whole intervention period was 91.1% (*n* = 15). For the training period in the living lab, the attendance rate reached 91.7% whereas 91.0% of all training sessions were attended during the training period at home. Both attendance rates were above the predefined 70% threshold rate, which was considered as acceptable adherence. Ten participants completed all planned 24 training sessions. Reasons for not attending training sessions were: holidays, being busy, work, family affairs, and temporary health issues (e.g., catching a cold). After the 8-week training intervention, the attrition rate of 28.6% (six drop-outs) was higher than the predefined expectable drop-out rate of 10%. Nevertheless, the sample size required for gaining valuable preliminary information remained well above the recommended 12 individuals. Three drop-out reasons were associated with technical problems and malfunctions of the training systems (software problems with “system crashes,” unstable IMU connections, inappropriate movement evaluation), which led to a decreased training motivation. In the other three drop-outs, the reasons for terminating the study were related to either personal or health issues (injury, sudden illness, family affairs).

During the intervention period, none of the 15 participants suffered any adverse events related to the study intervention, especially no falls or injuries during measurements or training. Moreover, no fear of falling during exergame training was reported. However, one participant reported to have experienced a “critical moment” where she almost fell because of swaying during a strength exercise in a lunge position. Light dizziness during training was reported by three participants (related to body rotations during dancing). Slight pain during training was stated by three participants whereas in one case, the pain was related to a foot injury which happened unrelated to the training. The other two participants reported slight knee and/or back pain.

**FIGURE 3:**
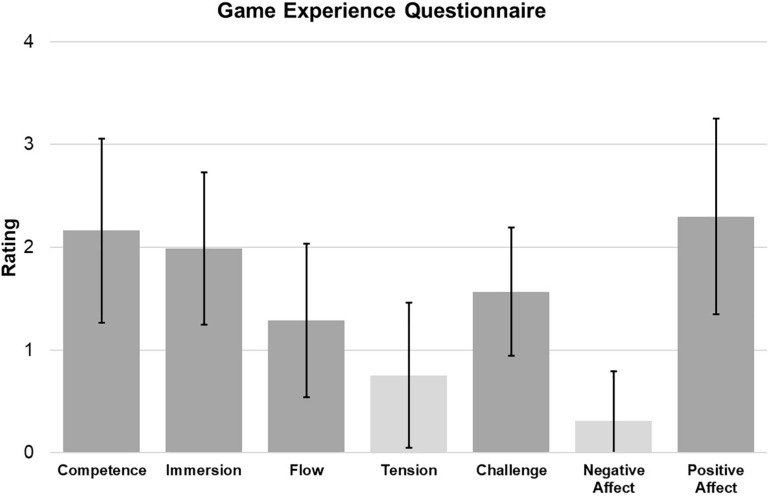
Primary outcome result of game experience. Data shown in the figure are means with standard deviations in each emotional category of the GEQ (*n* = 17). The two light-gray categories (tension, negative affect) have to be evaluated reversely which means a lower score is favorable. The GEQ ranges from 0 = “not at all” to 4 = “extremly”.

The SUS score was 70.6 ± 19.8 (*n* = 17) showing acceptable usability (a predefined 70-point score was considered acceptable for usability). In the additional question, asking the participants about their general opinion of the Active@Home exergame, the mean score was 3.2 ± 1.0 (on a scale from 0 = “I don’t like it” to 4 = “I like it a lot”). Figure 3 presents the results of the GEQ (*n* = 17), indicating positive game experience based on the above-average score of *positive affect* (2.3 ± 1.0) and the low scores of *negative affect* (0.3 ± 0.5) and *tension* (0.8 ± 0.7). The ratings for *competence* (2.2 ± 0.9) and *immersion* (2.0 ± 0.7) were around average whereas the scores of *challenge* (1.6 ± 0.6) and *flow* (1.3 ± 0.8) were slightly lower. Table 2 summarizes the main feedback of the participants and the observations of the supervisors during the training in the living lab setting.

**TABLE 2:** Primary outcome results: summary of usability protocol with supervisors’ observations and participants’ feedback.

**Criteria**	**Positive aspects**	**Negative aspects**
Functionality and interaction with the system	– Simple set up – Easy usable game composition	– Very specific movements/steps needed for step-based cognitive games (specific and strict step detecting algorithm) – Technical malfunctions of HDMI dongles
IMUs	– Comfortable to wear – Easy to attach (slap band) to wrists/ankles – Slap bands are easy to clean (e.g., from sweat) – Feasible navigation with the right hand IMU (“hand mouse”) – Battery can be easily charged	– Instable IMU connection (via Bluetooth) – Activation of the “hand mouse” in inappropriate situations (e.g., during dancing) – Suboptimal navigation for left-hander – Technical malfunctions of IMUs
Design	– Appealing game design – Pleasant and diverse music – Cute and funny cartoon-based instructor	– Virtual instructor could be more motivating (gestures as “thumps up”, comments etc.)
Training principles	– Real-time feedback while exercising (auditory and visual, positive and negative) – Performance score as feedback after exercising is motivating – High variability of exercises and levels	– Auditory feedback (sounds for “right” and “wrong”) could be louder in relation to background music – Feedback not always traceable – Partly inaccurate evaluation of movements
Exercises	– Clear structure of exercise levels – Entertaining quiz questions between strength exercises – Challenging strength and balance exercises (especially higher levels) – Exciting and challenging step-based cognitive games – Duration of the exercise is displayed – Helpful cues (arrows) to prepare the next movement	– Dance steps sometimes not perfectly instructed (difficult to reproduce) – Turns in dancing are difficult as no view on TV while turning – Instructions in English (difficult to understand for German-speaking participants) – Only frontal view of exercises (side view missing)
Emotions	– Motivation and fun (despite of technical malfunctions) – Curiosity about what is coming in the next levels – Happiness when seeing progress or achieving more performance points	– Frustration and displeasure because of technical issues and inaccurate evaluation of movements – Impatience with navigation

*IMU, inertial measurement unit.*

### Secondary Outcome: Effects of the Intervention

Table 3 summarizes median and variability values for the measures of secondary outcome.

**TABLE 3:** Secondary outcome results.

**Secondary Outcomes**	**Assessed by**	**Pre**	**Post**	***z***	***p***	***r***
**Physical functions**	**Gait analysis**					
	**Speed [m/s]**					
	ST walking DT walking DT costs [%]	1.41 (1.32; 1.49) 1.23 (1.13; 1.42) 9.4 (6.5; 18.3)	1.43 (1.23; 1.55) 1.34 (1.12; 1.44) 10.1 (2.5; 17.2)	−0.454 −1.590 −1.477	0.679 0.121 0.151	0.08 0.29 0.27
	**Cadence [steps/min]** ST walking DT walking DT costs [%]	119.4 (114.8; 120.7) 112.9 (103.1; 114.8) 6.8 (3.6; 12.1)	118.9 (111.3; 123.3) 113.3 (104.7; 117.3) 5.1 (3.2; 12.0)	−0.909 −1.704 −1.533	0.389 0.095 0.135	0.17 0.31 0.28
	**Stride length [m]** ST walking DT walking DT costs [%]	1.40 (1.35; 1.51) 1.30 (1.25; 1.52) 4.7 (0.1; 6.9)	1.41 (1.33; 1.47) 1.40 (1.25; 1.51) 3.1 (−1.6; 5.9)	−0.341 −1.022 −0.795	0.762 0.330 0.454	0.06 0.19 0.15
	**Minimal toe clearance [cm]** ST walking DT walking DT costs [%]	1.9 (1.5; 2.4) 2.0 (1.5; 2.7) −4.5 (−36.3; 2.4)	2.3 (1.9; 3.0) 2.4 (1.5; 3.1) 6.2 (−10.5; 16.4)	−2.158 −1.306 −0.852	0.030^∗^ 0.208 0.421	0.39 0.24 0.16
	
	**Extended Balance Test of SPPB**					
	Balance score	7 (6; 7)	7 (6; 7)	−0.857	0.488	0.16
	
	**Senior Fitness Test (SFT)**					
	30 s chair rises test 2 min stepping test	15 (13; 20) 94 (74; 114)	18 (15; 22) 100 (79; 113)	−1.603 −1.137	0.110 0.270	0.29 0.21
	
	**Y-Balance Test (YBT)**					
	YBT score [%]^+^	76.8 (73.7; 85.2)	87.1 (73.9; 91.1)	−1.836	0.074	0.43

**Cognitive functions**	**Divided Attention Test of TAP**					
	RT auditory [ms] RT visual [ms] Errors Omissions	624 (577; 675) 834 (798; 980) 3 (1; 5) 2 (1; 3)	648 (617; 691) 892 (813; 1012) 3 (1; 5) 2 (0; 3)	−0.057 −0.454 −0.466 −0.051	0.966 0.679 0.730 0.953	0.01 0.08 0.09 0.01
	
	**Trial Making Test (TMT)** **TMT A** Time [s] Errors **TMT B** Time [s] Errors	38.3 (35.1; 46.5) 0 (0; 0) 85.9 (72.0; 107.8) 0 (0; 2)	33.6 (31.2; 50.0) 0 (0; 0) 85.5 (70.5; 109.0) 1 (0; 2)	−0.625 −1.000 −0.511 −0.520	0.561 1.000 0.639 0.656	0.11 0.18 0.09 0.09
	
	**Wechsler Memory Scale-Revised (WMS-R)**					
	Digit forward task Digit backward task	6 (6; 8) 5 (5; 6)	8 (6; 9) 6 (6; 7)	−2.859 −0.890	0.002^∗^ 0.434	0.52 0.16
	
	**Victoria Stroop Test (VST)** **VST 1** Time [s] Errors **VST 2** Time [s] Errors **VST 3** Time [s] Errors	13.2 (12.4; 15.2) 0 (0; 0) 16.5 (14.1; 19.2) 0 (0; 0) 25.0 (22.0; 29.0) 0 (0; 1)	12.5 (11.4; 14.5) 0 (0; 0) 15.2 (14.6; 16.1) 0 (0; 0) 22.8 (19.0; 27.2) 0 (0; 1)	−2.727 −0.707 −1.789 −1.414 −1.533 −1.023	0.004^∗^ 0.750 0.075 0.500 0.135 0.328	0.50 0.13 0.33 0.26 0.28 0.19

**Cortical activity**	**Resting state EEG**					
	Peak alpha frequency [Hz] Center of gravity [Hz]	9.3 (8.7; 9.5) 8.8 (8.5; 9.5)	9.7 (9.0; 9.8) 9.2 (8.8; 9.5)	−1.104 −1.682	0.375 0.105	0.25 0.36
	Alpha spectral power [μV^2^]	23.4 (20.1; 39.2)	27.8 (19.8; 63.9)	−1.955	0.055	0.44

*Data are median values (interquartile range). *n* = 15 (^+^expect of the YBT with *n* = 9). ^∗^*p* < 0.05, *p*-values are exact sig. two-tailed. Pre-post differences were evaluated using Wilcoxon signed-rank test. For effect size r, *r* = 0.10 indicates a small effect, *r* = 0.30 a medium effect, and *r* ≥ 0.50 a large effect (Cohen, 1988). ST, single-task. DT, dual-task. DT costs are calculated as (ST – DT)/ST × 100. SPPB, Short Physical Performance Battery. TAP, Test of Attentional Performance. RT, reaction time.*

For gait analysis, the minimal toe clearance under single-task walking condition increased significantly (*z* = −2.158, *p* = 0.030, *r* = 0.39) comparing pre- and post-measurement. Considering the balance assessments, nine participants reached 20 s or more for the single-leg stance and, thus, were eligible for the YBT test. A medium to large effect size (*z* = −1.836, *p* = 0.074, *r* = 0.43) was evident for these individuals with a tendency of higher dynamic balance scores after the intervention. In the digit forward task of the WMS-R, a significant increase in the performance score was found (*z* = −2.859, *p* = 0.002, *r* = 0.52) comparing pre- and post-measurement. Furthermore, reaction time in the first task of the VST (VST 1) was significantly faster (*z* = −2.727, *p* = 0.004, *r* = 0.50) after the training intervention. No significant changes in resting state EEG were found for the pre-post-comparison. However, a medium to large effect size was evident for the alpha spectral power (*z* = −1.955, *p* = 0.055, *r* = 0.44) with a tendency of increased spectral power in the individual alpha frequency band after training.

### Other Outcome Results

Participants rated their training motivation on average with 4.2 ± 0.7 (*n* = 20). The motivation for the training in the living lab was 4.2 ± 0.6 (*n* = 20) and the motivation for the home-based training was 4.1 ± 0.8 (*n* = 17). The average rating of participants’ perceived exertion for Tai Chi-inspired exercises was 11.9 ± 1.5 (*n* = 20) and for dance exercises 11.0 ± 1.5 (*n* = 20). Both ratings reflect an intensity on the 20-point Borg scale that corresponds from “fairly light” (11) to “somewhat hard” (13). The average HR during Tai Chi-inspired training was 60 ± 10% of the individual maximal HR, the average HR during dancing training was also 61 ± 11% of the HR_*max*_.

## Discussion

The primary aim of this study was to evaluate the feasibility and usability of an in-home exergame training for older adults. Furthermore, potential training-related adaptations, both in physical and cognitive aspects of functioning, were explored. In summary, the study results showed a general feasible and well-accepted exergame that indicates to endorse measurable training effects in certain physical and cognitive functions.

### Feasibility and Usability

The conclusion of a feasible training system is based on the high adherence rate of over 90%. Interestingly, the adherence rates for the training periods in the living lab and at home were almost the same (91.7% and 91.0%, respectively). Therefore, the presence of a supervisor during the living lab training sessions might not have promoted the training adherence compared to the in-home setting. Although, a widespread opinion is that social interaction increases training motivation in older adults (Smith et al., 2017). The high adherence rate found in this study is in line with the result of the first exploratory study testing the newly developed exergame in a laboratory setting. Furthermore, previous studies showed high training attendance in older adults using exergames (De Bruin et al., 2011; Wuest et al., 2014). Exergame interventions often showed higher adherence compared to standard fall prevention exercises (Sjösten et al., 2007; De Bruin et al., 2011; Wuest et al., 2014). One reason might be the high motivational potential and playfulness of exergames leading to a captivating and entertaining training (Vaziri et al., 2016; Chesham et al., 2017; Valenzuela et al., 2018). Accordingly, the motivation ratings in this study were equally high for the training in the living lab and at home. In general, high motivation seems to be crucial for the success of training interventions as motivation might lead to a high training compliance and, therefore, training related benefits can reach their full potential (van Het Reve et al., 2014). On the other hand, the attrition rate in this study was higher than the predefined rate (Nyman and Victor, 2012). Even after subtracting the drop-outs resulting from reasons not related to the training system itself (e.g., due to health issues), attrition was still higher than the predefined rate. Although, the drop-out rate is about 10% on average in exergame studies (Skjæret et al., 2016), it can reach up to 40% in individual studies (Larsen et al., 2013). Exergames might be more prone to technical issues leading, in some cases, to higher drop-out rates. Interestingly, the drop-outs were not related to safety concerns of participants (e.g., fear of falling during training) or even injuries related to exercising. In general, participants reported no safety issues beside of isolated cases of light insecurities or dizziness. It has to be considered, that the Active@Home exergame was developed for healthy older adults living independently at home. Exercises might have to be adapted if the exergame should target clinical populations.

Despite the rather high attrition rate, the usability of the exergame was rated with an acceptable score (SUS score of 70.6/100) and a general positive feedback was given by the participants. They were satisfied with the game story, the virtual instructor, and the training content including diverse and challenging exercises and cognitive stimulation. Comparing to existing literature, exergames in general have been shown to be well accepted and usable for healthy older adults (Gerling and Mandryk, 2014; Nawaz et al., 2016; Vaziri et al., 2016). Additionally to the participants’ feedback, supervisors’ observation during the training period in the living lab gave further insights in the general positive user experiences e.g., easy usable system set up and simple attachment of the IMUs. However, some discrepancies were evident comparing the supervisors’ observations and the participants’ positive feedback. Despite the high rated usability, difficulties in using the technology-based system (e.g., navigating through the game) have been observed and help was occasionally requested by participants. Previous experiences with technology might have been beneficial as for example the navigation by the hand IMU could be partly related to previous experiences with other navigation systems, e.g., the computer mouse. The most threatening aspect for the usability of the Active@Home exergame was related to technical malfunctions of the system. Participants often were frustrated and disappointed when the IMU connections were unstable or movement detection was incorrect. Therefore, a technology-based training system with flawless functionality and age-appropriate design may be crucial considering the potentially restricted technology knowledge of older adults and the independent use of the training system at home (Brach et al., 2012; Bleakley et al., 2015; Vaziri et al., 2016).

The emotional reactions evident in the observations were also reflected in the results of a questionnaire assessing emotions and game experience (GEQ). Compared to other exergame studies using this questionnaire (Gerling et al., 2011; Liukkonen et al., 2015; Merriman et al., 2018), *positive affect* was rated slightly lower whereas *tension* was rated slightly higher in this study. Nevertheless, the emotional experience during training with the Active@Home exergame can be interpreted as general positive considering ratings of positive emotions above average (e.g., feeling captivated and pleased) and a low rating of negative affect (e.g., feeling tensed and annoyed). Emotions related to challenge and flow were rated rather low which might be explained by inappropriate training load and by technical problems interrupting the game flow. In sum, the Active@Home exergame provided mainly positive emotional experiences which might be linked to the high training motivation as well as the high training adherence.

### Effects of the Intervention

Our study results showed a significant improvement in minimal toe clearance under single-task walking leading to higher “foot lifting” after training which might be related to a decreased risk of tripping and falling in older adults (Barrett et al., 2010). Improvements in gait parameters after exergame training have been found in several previous studies, mainly under dual-task walking (Pichierri et al., 2012; Schättin et al., 2016; Wang et al., 2017). These effects on multitasking capabilities are discussed to be due to the characteristics of exergame training targeting the physical-cognitive interplay (Segev-Jacubovski et al., 2011; Beurskens and Bock, 2012). Even though the Active@Home exergame included training of physical and cognitive functions, no significant improvements in gait parameters under dual-task walking have been found in this study. Regarding the other assessed physical functions, the statistical analysis did not reveal any significant changes after the training intervention. Nevertheless, a medium to large effect size was found for dynamic balance assessed by the YBT despite the small sample size due to restricted participant eligibility. In contrast, measures of static balance assessed by the SPPB showed no improvement which is in line with the results of the first Active@Home exergame study. We might conclude that the Active@Home exergame has a stronger focus on training of dynamic balance or that the SPPB is not sensitive enough to assess balance differences in an upper range of static balance performance. Moreover, optimal training load in a moderate to vigorous exercise intensity might be questioned considering the subjective ratings of perceived exertion during trainings (Borg scale ratings) and objectively assessed training intensity (heart rate measurements) (Nelson et al., 2007; Chodzko-Zajko et al., 2009). The suboptimal training load and the rather short training period might have restricted the training impact on additional physical outcomes. Therefore, further studies should focus on ensuring optimal training intensity as well as extended training duration.

In the cognitive tests, study results showed a significant change in the short-term attentional span (i.e., digit forward task of WMS-R), whereas an improvement in general attentional focus and information processing speed might be assumed. Accordingly, information processing speed in another task (i.e., VST 1) significantly improved after the training intervention resulting in faster reaction times. These positive effects might be attributed to the cognitive training components and the physical-cognitive interaction required in the exergame training. Similarly, recent studies showed significant enhancement of cognitive abilities including information processing speed after exergame training in older adults (Maillot et al., 2012; Schoene et al., 2013; Ogawa et al., 2016; Stanmore et al., 2017). Interestingly, a cognitive training alone (e.g., computer-based training) without elements of physical activity seems to have only small or none effects on cognitive functions (Lampit et al., 2015) leading to the suggestion that exergames have a greater impact on cognition than sole computer-based training. Nevertheless, no significant changes were evident for executive functions as mental flexibility and inhibition. As mentioned before, the rather short intervention period might have also limited the training effects on cognitive functions.

Our study results of the neuronal activity measurements during resting state EEG showed a medium to large effect size for alpha spectral power with a tendency to a power increase after the training intervention. A variety of studies reported age-related changes in cortical oscillatory activity (Klimesch, 1999), often summarized as age-related “slowing” and described as an EEG power increase in the slow frequency ranges (<7 Hz) and a decrease in higher frequencies (>7 Hz), especially in posterior brain regions (Rossini et al., 2007; Babiloni et al., 2016). Moreover, the individual alpha frequency peak is known to decrease in the later part of lifespan (Klimesch, 1999). A positive correlation between EEG power within the alpha frequency band and global cognitive status in healthy as well as in impaired older adults is considered (Klimesch, 1999; Vecchio et al., 2013). Our study results support the suggestion that the age-related “slowing” in cortical oscillatory activity can be counteracted with physical and cognitive training (Babiloni et al., 2016). However, we found no significant changes in measurements of the IAF.

### Limitations

As this is a pilot study with primary focus on feasibility and usability outcomes, secondary results have to be interpreted with caution (Arain et al., 2010; Thabane et al., 2010). The small sample size might have limited the detection of significant training effects comparing pre- and post-measurements. Nevertheless, pilot studies are especially performed to generate preliminary data to allow system and protocol development for future studies. Based on the “rule of 12”, we can be confident about our values (Moore et al., 2011). Moreover, the lack of a control group might have limited further explorative analyses of training effects comparing pre- and post-measurements. As next step, a randomized controlled trial should be conducted to carefully evaluate the effectiveness of the Active@Home exergame. Moreover, in future studies, the training period should be extended to increase the potential for training improvements.

## Conclusion

Our study results showed a general high feasibility and usability of the adapted Active@Home exergame and an overall positive emotional game experience in a living lab as well as in a home-based setting. These results lead to the conclusion that the presence of a supervisor might not be a crucial factor regarding training compliance and motivation. Furthermore, this study demonstrated that older adults were able to use the exergame in an in-home setting. However, simplicity and flawless technical functionality of the training system should be a mandatory development consideration. Moreover, a rather high variability in participants’ general feedback about the exergame training was present reflecting the individual experiences and preferences. Thus, we might conclude that the Active@Home exergame is a general feasible and usable in-home training satisfying the needs and requirements of at least a part of the older population. Additionally, results of physical and cognitive testing indicate that the exergame might have positive influence on crucial functions in older adults. However, the efficacy has to be evaluated in a future randomized controlled trial.

## Data Availability Statement

The raw data supporting the conclusions of this manuscript will be made available by the authors, without undue reservation, to any qualified researcher upon request.

## Ethics Statement

This study was carried out in accordance with the recommendations of the ETH Zürich Ethics Committee (Zurich, Switzerland). All subjects gave written informed consent in accordance with the Declaration of Helsinki. The protocol was approved by the ETH Zürich Ethics Committee (EK 2018-N-07).

## Author Contributions

MA and MT developed the research question under the lead of EB. MA and MT established the concept and design while AS and EB acted as methodological council. MA and MT conducted data acquisition, analysis and interpretation of the results with edition and improvement by AS and EB. FG performed EEG data analysis and interpretation of the EEG results, which was edited and improved by MA, AS, and EB. MA and MT produced a first version of the manuscript. AS, FG, and EB substantially revised the manuscript to bring it to its current version. All authors have read and approved the final manuscript.

## Conflict of Interest

The authors declare that the research was conducted in the absence of any commercial or financial relationships that could be construed as a potential conflict of interest.

## Footnotes

1 FunctionalMovement.com
